# Exposure of domestic mammals to West Nile virus during an outbreak of human encephalitis, New York City, 1999.

**DOI:** 10.3201/eid0704.010424

**Published:** 2001

**Authors:** N Komar, N A Panella, E Boyce

**Affiliations:** Centers for Disease Control and Prevention, P.O. Box 2087, Fort Collins, CO 80522, USA. nck6@cdc.gov

## Abstract

We evaluated West Nile (WN) virus seroprevalence in healthy horses, dogs, and cats in New York City after an outbreak of human WN virus encephalitis in 1999. Two (3%) of 73 horses, 10 (5%) of 189 dogs, and none of 12 cats tested positive for WN virus-neutralizing antibodies. Domestic mammals should be evaluated as sentinels for local WN virus activity and predictors of the infection in humans.


### Exposure of Domestic Mammals to West Nile Virus during an Outbreak of Human Encephalitis, New York City, 1999

**Nicholas Komar,* Nicholas A. Panella,* and Edward Boyce†**
*Centers for Disease Control and Prevention, Fort Collins, Colorado, USA; and
†New York City Department of Health, New York, New York, USA

We evaluated West Nile (WN) virus seroprevalence in healthy horses, dogs, and cats in New York City after an outbreak of human WN virus encephalitis in 1999. Two (3%) of 73 horses, 10 (5%) of 189 dogs, and none of 12 cats tested positive for WN virus-neutralizing antibodies. Domestic mammals should be evaluated as sentinels for local WN virus activity and predictors of the infection in humans.

An outbreak of West Nile (WN) viral encephalitis in New York City during the summer of 1999 resulted in numerous human cases and several deaths of elderly patients (1). WN virus, a mosquito-borne flavivirus, had not previously been recognized in New York City; therefore, the city's public health system had no surveillance guidelines in place before the outbreak. Such guidelines require basic epidemiologic and ecologic data. Entomologic studies (2), avian seroprevalence studies (3), and a human serosurvey (4) have been completed. This report presents seroprevalence data in New York City horses, dogs, and cats and discusses the potential of these animals as sentinels for human infection.

### The Study

Serum samples were collected, by standard procedures, from healthy stabled horses and from stray dogs at animal shelters in each of the five New York City boroughs (counties) from September 15 to November 1, 1999. Additionally, samples from healthy, privately owned dogs and cats were obtained from veterinary practices in Queens and neighboring communities to the east in Nassau County during routine clinic visits. Serum samples were processed and tested for neutralizing antibodies to WN virus and St. Louis encephalitis (SLE) virus (a closely related flavivirus) as in a previous study (3), except that only samples positive for WN virus-neutralizing antibodies were also tested for SLE virus-neutralizing antibodies. Samples with reciprocal 90% neutralization titers of $\geq$10 were considered positive for flavivirus infection. However, a fourfold difference in titer for one of the two flaviviruses was required for a specific flavivirus to be considered an etiologic agent of the infection. Approximate ages were recorded for each animal sampled.

Neutralizing antibodies to WN virus were detected in horses and dogs in several boroughs (Tables 1, 2). Overall, 2 (3%) of 73 horses and 10 (5%) of 189 dogs, tested positive for WN virus-neutralizing antibodies. All 12 cats tested negative. Reciprocal titers were 80 to $\geq$ 320 for dogs and $\geq$ 320 for both positive horses. In all cases, WN antibody titers were at least fourfold higher than SLE titers (data not shown). Thus, all these infections were attributed to WN virus, and none were attributed to SLE virus. All but one of the 12 infections were in animals from the Queens and Bronx boroughs. The seroprevalence in dogs from these two counties was determined within several age categories (Table 3) to examine whether the pattern of seropositivity in relation to age resembled enzootic or epizootic transmission. More dogs were exposed in the youngest age category than in older categories (p = 0.026, Fisher exact test).

Dogs from Queens were further analyzed for the effect of stray status versus pet status (data not shown). Strays had

Address for correspondence: Nicholas Komar, Centers for Disease Control and Prevention, P.O. Box 2087, Fort Collins, CO 80522, USA; fax: 970-221-6476; e-mail: nck6@cdc.gov

Table 1. West Nile virus-neutralizing antibodies in horses, by county

| County | Total tested | No. pos. (% [95% CI]) |
|---|---|---|
| Queens | 18 | 1 (5.6 [0.1-27.3]) |
| Bronx | 19 | 1 (5.3 [0.1-26.0]) |
| Richmond | 6 | 0 |
| Kings | 10 | 0 |
| New York | 20 | 0 |
| Total | 73 | 2 (2.7 [0.3-9.5]) |

CI: confidence interval.

Table 2. West Nile virus-neutralizing antibodies in dogs, by county

| County | Total tested | No. pos. (% [95% CI]) |
|---|---|---|
| Queens | 55 | 6 (10.9 [4.1-22.2]) |
| Bronx | 25 | 3 (12.0 [2.5-31.2]) |
| Richmond | 20 | 1 (5.0 [0.1-24.9]) |
| Kings | 22 | 0 |
| New York | 21 | 0 |
| Nassau | 46 | 0 |
| Total | 189 | 10 (5.3 [2.6-9.5]) |

CI: confidence interval.

Table 3. West Nile virus-neutralizing antibodies in dogs from Queens and the Bronx, by age

| Age | Total tested | No. pos. (% [95% CI]) |
|---|---|---|
| <2 yrs | 43 | 7 (16.3 [6.8-30.7]) |
| 2-4 | 20 | 1 (5.0 [0.1-24.9]) |
| >4 yrs | 16 | 1 (6.3 [0.1-30.2]) |
| Unknown | 1 | 0 |
| Total | 80 | 9 (11.2 [5.3-20.3]) |

CI: confidence interval.

WN virus seroprevalence of 15% (3 of 20), whereas pets had 8.6% (3 of 35). Although strays had higher seroprevalence than pets, the difference was not significant ($p = 0.657$, Fisher exact test).

### Conclusions

We present serologic evidence of WN virus infection in New York City horses and dogs in 1999. In epidemiologic studies in the Middle East and Africa, WN virus-infected horses and dogs have been frequently detected in serologic surveys (5,6). Severe disease caused by WN virus in dogs is unknown, but epizootics of WN encephalomyelitis in horses have been described in several countries (7-9). Such an epizootic in New York horses (10) was observed concurrently with our study, with cases clustered in eastern Suffolk County. At least one equine case of WN encephalitis occurred close to New York City in Belmont, Nassau County.

Cats were not adequately sampled to determine valid seroprevalence figures. We were unable to find reference to any other serologic surveys in cats for WN virus antibodies. Cats appear to be "refractory" to infection with SLE virus (11), a common North American flavivirus, because they do not generate a humoral immune response after experimental infection and have not been found to develop antibodies in field serosurveys. Cats do develop antibodies to Powassan virus, another North American flavivirus (12). WN virus was isolated from brain tissue of a cat with neurologic symptoms in New Jersey in 1999 (13). Detectable levels of neutralizing antibodies did not develop in this cat before euthanasia (Centers for Disease Control and Prevention, unpub. data). The role of cats in the epidemiology of WN virus in the New York City region has yet to be determined.

The outbreak of WN virus in New York City and vicinity in 1999 was the first recorded instance of natural WN virus activity in the New World. The definitive date of WN virus introduction to the United States has not yet been established. We evaluated WN seroprevalence in dogs of different ages to determine if prevalence of infection increased with age, which would suggest that WN virus transmission in New York City dogs may have occurred before 1999. However, seroprevalence appeared weighted toward younger animals. This may reflect a skewing of the younger age groups by stray dogs, which probably are more likely to be exposed to biting mosquitoes. Our results support the hypothesis that WN virus was introduced in 1999, although they do not prove it.

Both dogs and horses are presumably dead-end hosts in the WN virus transmission cycle, which involves mosquito vectors and avian reservoir hosts (13). Although infection studies in horses with the New York strain of WN virus have not yet been published, such studies are expected to reaffirm the findings of previous studies (14,15) that these animals develop very low, ephemeral viremia insufficient for infecting mosquitoes (J. Lubroth, R. Bowen, pers. comm.). An infection study in dogs using an African strain of WN virus yielded similar findings (6). Thus, the presence of horses and dogs seropositive for WN virus does not necessarily indicate a human risk for WN infection. In fact, these horses and dogs may offer protection (zooprophylaxis) because they may divert potentially infectious bites of mammalophilic mosquitoes away from human hosts.

Further evidence of a zooprophylactic effect from pet dogs and horses is that seroprevalence in humans was approximately 2.5% in the epicenter of the outbreak in northeastern Queens (4), substantially lower than seroprevalence in dogs or horses in Queens (Table 1). The higher seroprevalence in these species indicates greater exposure to infectious mosquito bites. Whether this exposure in dogs and horses is due simply to increased time spent outdoors at night (when *Culex* species mosquitoes are feeding), greater attractiveness to blood-seeking mosquitoes, or other factors is unknown. Nonetheless, the finding that WN virus antibodies are readily detected in dogs and horses suggests a possible role for these animals as sentinels for human risk due to WN virus transmission.

Sentinels for human infection are frequently used in public health programs for monitoring arbovirus. Typically chickens and wild birds (e.g., house sparrows) have been used as sentinels for mosquito-borne monitoring programs in the United States (16-18) because of the role of birds as primary reservoir hosts for many of these viruses, including WN virus. However, whereas infection in birds may signal enzootic virus activity in birds, it may provide a less effective warning for risk in mammals. Such risk occurs when certain species of mosquitoes that act as "bridge" vectors to mammals become abundant. Thus, using nonhuman mammals as sentinels for human infection may have merit for effective risk management programs. Indeed, horses have been used as public health sentinels for both eastern and western equine encephalitis viruses, mosquito-borne agents of human encephalitis in the United States (19,20).

In summary, the finding that approximately 5% of horses and 10% of dogs were infected with WN virus in certain boroughs within New York City (The Bronx and Queens) in 1999 suggests a possible role for these domestic animals as sentinels for WN virus infection in humans. Seroconversions in these animals may signal increased risk for WN virus transmission to other mammals, including humans. An age-stratified analysis of seroprevalence data in dogs from boroughs of Queens and the Bronx found no evidence of a long-term pattern of infection in dogs, suggesting that all infections probably occurred as a result of the 1999 outbreak.

### Acknowledgments

We thank Joseph Charros, Dennis Farrell, and Frank Borzio for assisting with blood sample collection; Stephen Dusza for providing statistical analyses; Joseph Burns for assisting with dog specimen collection in Queens and Nassau County; and Iqbal Poshni and Allan Goldberg for assisting with dog specimen collection in New York City.

Dr. Komar is the vertebrate ecologist for the Centers for Disease Control and Preventions' Arbovirus Diseases Branch, Division of Vector-Borne Infectious Diseases, Fort Collins, Colorado. His major research interest is in understanding the role of vertebrate hosts in arbovirus transmission cycles.

### References

1. Centers for Disease Control and Prevention. Update: West Nile virus encephalitis—New York, 1999. MMWR Morb Mortal Wkly Rep 1999;48:944-6, 955.
2. Nasci RS, White DJ, Stirling H, Oliver J, Daniels TJ, Falco RC, et al. West Nile virus isolates from mosquitoes in New York and New Jersey, 1999. Emerg Infect Dis 2001;7:626-30.

3. Komar N, Panella NA, Burns J, Dusza S, Mascarenhas TM, Talbot T. Serologic evidence for West Nile virus infection in birds in the New York City vicinity during an outbreak in 1999. Emerg Infect Dis 2001;7:621-5.
4. Centers for Disease Control and Prevention. Serosurveys for West Nile virus infection—New York and Connecticut counties, 2000. MMWR Morb Mortal Wkly Rep 2001;50:37-9.
5. Hayes CG. West Nile fever. In: Monath TP, editor. The arboviruses: epidemiology and ecology. Vol V. Boca Raton (FL): CRC Press; 1998. p. 59-88.
6. Blackburn NK, Reyers F, Berry WL, Shepherd AJ. Susceptibility of dogs to West Nile virus: a survey and pathogenicity trial. J Comp Pathol 1989;100:59-66.
7. Joubert L, Oudar J, Hannoun C, Beytout D, Corniou B, Guillon JC, et al. Epidemiology of the West Nile virus: study of a focus in Camargue. IV. Meningo-encephalomyelitis of the horse. Annals of the Institute Pasteur (Paris) 1970;118:239-47.
8. Tber AA. West Nile fever in horses in Morocco. Bulletin of the Office International des Epizooties;1996;108:867-9.
9. Cantile C, Di Guardo G, Eleni C, Arispici M. Clinical and neuropathological features of West Nile virus equine encephalomyelitis in Italy. Equine Vet J 2000;32:31-5.
10. Office International des Epizooties. West Nile fever in the United States of America in horses. Disease Information 1999;12:150-1.
11. McLean RG, Bowen GS. Vertebrate hosts. In: Monath TP, editor. St. Louis encephalitis. Washington: American Public Health Association; 1980. p. 381-450.
12. Keane DP, Parent J, Little PB. California serogroup and Powassan virus infection of cats. Can J Microbiol 1987;33:693-7.
13. Komar N. West Nile viral encephalitis. Rev Sci Tech 2000;19:166-76.
14. Taylor RM, Work TH, Hurlbut HS, Rizk F. A study of the ecology of West Nile virus in Egypt. Am J Trop Med Hyg 1956;5:579-620.
15. Schmidt JR, El Mansoury HK. Natural and experimental infection of Egyptian equines with West Nile virus. Ann Trop Med Parasitol 1963;57:415-27.
16. McLean RG, Mullenix J, Kerschner J, Hamm J. The house sparrow (*Passer domesticus*) as a sentinel for St. Louis encephalitis virus. Am J Trop Med Hyg 1983;32:1120-9.
17. Day JF, Stark LM. Avian serology in a St. Louis encephalitis epicenter before, during, and after a widespread epidemic in south Florida, USA. J Med Entomol 1999;36:614-24.
18. Reisen WK, Lundstrom JO, Scott TW, Eldridge BF, Chiles RE, Cusack R, et al. Patterns of avian seroprevalence to western equine encephalomyelitis and Saint Louis encephalitis viruses in California, USA. J Med Entomol 2000;37:507-27.
19. Goldfield M, Sussman O, Black HC, Horman JT, Taylor BF, Carter WC, et al. Arbovirus infection of animals in New Jersey. J Am Vet Med Assoc 1968;153:1780-7.
20. Potter ME, Currier RW, Pearson JE, Harris JC, Parker RL. Western equine encephalomyelitis in horses in the Northern Red River Valley, 1975. J Am Vet Med Assoc 1977;170:1396-9.
